# Study on the function of *TTG1* gene in *Camellia oleifera*

**DOI:** 10.3389/fpls.2025.1612606

**Published:** 2025-08-26

**Authors:** Chengcheng Xiang, Lizhi Xiao, Hongmei Tao, Jie Cao, Jun Yuan, Weihao Wang, Yao Jiang

**Affiliations:** ^1^ Key Laboratory of Cultivation and Protection for Non-Wood Forest Trees, Ministry of Education, Central South University of Forestry and Technology, Changsha, China; ^2^ Key Laboratory of Non-Wood Forest Products of State Forestry Administration, Central South University of Forestry and Technology, Changsha, China; ^3^ College of Food Science and Engineering, Shanxi Agricultural University, Jinzhong, China

**Keywords:** *Camellia oleifera*, TTG1, trichomes, anthocyanins, fatty acids

## Abstract

*Camellia oleifera* is an economically important woody oil crop in China, where seed oil quality and yield are critical determinants of commercial value. Although the WD40-repeat transcription factor *TTG1* is known to regulate plant secondary metabolism and development, its specific functions in *C. oleifera* remain uncharacterized. In this study, we isolated the *CoTTG1* gene from *C. oleifera*, which encodes a nuclear-localized protein (molecular weight 38.38 kDa, pI 5.0) sharing 99.71% sequence identity with *Camellia japonica TTG1*. Heterologous expression resulted in: (1) significantly increased leaf trichome density (up to 114 trichomes/50 mm²); (2) enhanced seed anthocyanin accumulation (199–318% increase); and (3) substantial alterations in fatty acid composition, including 79% elevation in oleic acid (C18:1), 113% increase in gondoic acid (C20:1), 35% reduction in both linolenic (C18:3) and palmitic acids (C16:0), and 87% decrease in erucic acid (C22:1). Molecular analyses revealed that *CoTTG1* mediates these phenotypic changes through upregulation of trichome development-related genes (*AtETC1*, *AtGL1*, *AtCPC*), anthocyanin biosynthesis genes (*AtF3’H*, *AtLDOX*, *AtUFGT*), and lipid metabolism genes (*AtLACS8*, *AtSAD*). These findings demonstrate the pleiotropic regulatory roles of *CoTTG1* in controlling key agronomic traits, establishing it as a valuable molecular target for genetic improvement of *C. oleifera*.

## 1 Introduction

*Camellia oleifera* Abel. is an evergreen shrub or small tree that thrives in subtropical regions. The tea oil obtained from its seeds is popular because of its high nutritional content and medicinal value (Gao et al., 2011; Yang et al., 2015). As an important oil crop, *C. oleifera* has gained high reputation and has been widely planted in many countries, including China, the Philippines, India, Brazil, and South Korea, demonstrating its significant global impact and economic value (Lee and Yen, 2006; Wang et al., 2013). Tea oil, known as “Oriental olive oil”, is rich in monounsaturated fatty acids that are beneficial to human health (Lee et al., 2007; Wang et al., 2013). The main active flavonoids in *C. oleifera* have been widely used in medicine, particularly in antithrombotic, anti-myocardial ischemia, and anti-dementia treatments. Tea seed oil shows great potential in promoting human health (Lee et al., 2007; Liu et al., 2014; Shen et al., 2017).

The name WD40 is derived from the conserved WD dipeptide and the length of about 40 amino acid residues in a single repeat sequence. The WD40 protein is characterized by a core residue of 40–60 amino acid sequences, containing a glycine-histidine pair (GH) at the N-terminus and a tryptophan-aspartate pair (WD) at the C-terminus (Xu and Min, 2011; Hu et al., 2017). The WD40 protein motif is typically folded into a β-propeller domain repeat sequence, acting as a scaffold for various protein-protein interactions and forming functional complexes (Fang et al., 2019). Growing evidence shows that WD40 proteins play roles in signal transduction, chromatin assembly, cytoskeleton organization, cell division, flowering and abiotic stress response (Hu et al., 2020; Tan et al., 2021). One of the most well-known functions of WD40 proteins is to regulate anthocyanin/proanthocyanidin biosynthesis as part of the MYB-bHLH-WD40 (MBW) complex.

*TTG1* is a WD40-repeat protein conserved across land plants, containing multiple WD40 repeat domains. Previous studies have demonstrated the functions of *TTG1* in various plants, confirming its key roles in growth, development, and stress response (Walker et al., 1999; Serna and Martin, 2006). In *Arabidopsis thaliana*, the *TTG1* transcription factor plays pleiotropic roles, regulating anthocyanin biosynthesis, pigment accumulation, trichome development, seed coat mucilage formation, and stress resistance. *TTG1* interacts with R2R3-MYB and basic helix-loop-helix (bHLH) transcription factors to form MYB-bHLH-WD40 (MBW) complexes. These complexes precisely regulate downstream gene expression, controlling cell fate determination including trichome initiation (Xu et al., 2015; Golz et al., 2018). In *A. thaliana*, *TTG1* binds to the R2R3-MYB transcription factor *GLABRA1* (*GL1*) and bHLH factors *GLABRA3* (*GL3*) or *ENHANCER OF GLABRA3* (*EGL3*) to form MBW complexes that activate *GLABRA2* (*GL2*) expression, regulating epidermal cell differentiation (Schiefelbein, 2003).

These findings, together with earlier work (Galway et al., 1994; Windsor et al., 2000; Zhang, 2003; Baudry et al., 2004; Aoyama et al., 2012; Xu et al., 2014, 2015), demonstrate *TTG1*’s central position in plant developmental networks. Recent work further identified *OsTTG1* as a key WD40 regulator of anthocyanin biosynthesis in rice through MBW complex formation (Yang et al., 2021). In this study, we characterized *CoTTG1* through PCR, qPCR, gas chromatography, and spectrophotometry. The nuclear-localized *CoTTG1* protein, when overexpressed in *A. thaliana*, significantly increased leaf trichome density, promoted seed anthocyanin accumulation, and altered seed fatty acid composition.

## 2 Materials and methods

### 2.1 Plant materials and culture conditions

In this study, we used the *C. oleifera* cultivar ‘Huashuo’ grown in the experimental field of Central South University of Forestry and Technology (geographical location: 28°05′N, 113°02′E) as plant materials. Twenty healthy *C. oleifera* trees with uniform growth vigor were randomly selected. Seeds were collected from four cardinal directions (northeast, southeast, northwest, and southwest) during five developmental stages (Song et al., 2021): 13 July (globular to heart-shaped embryo, G1 stage); 5 August (torpedo to cotyledon-stage embryo, T3 stage); 10 September (mature cotyledon embryo, M5 stage); 12 October (early mature fruit); 4 November (fully mature fruit). A total of 60 seeds were collected, with 3 seeds per tree per developmental stage.

The wild-type *A. thaliana* (ecotype Columbia-0) was used as transgenic receptor material. Seeds were surface-sterilized, vernalized at 4°C for 2 days in darkness, then sown evenly in growth substrate (vermiculite: nutrient soil = 1:1, v/v) and covered with transparent plastic wrap. Plants were grown under controlled conditions: 20-22°C, 100-150 μmol m^-2^ s^-1^ photosynthetic photon flux density, and 14-hr photoperiod. After 7 days of germination, seedlings were transplanted to new pots (1–2 plants per pot). When plants reached the rosette stage (diameter ≥5 cm), experimental treatments or phenotypic analyses were conducted.

### 2.2 Total RNA extraction and cDNA synthesis.

Total RNA was extracted from the leaves, flowers, and seeds of *C. oleifera* ‘Huashuo’ using the FastPure Universal Plant Total RNA Isolation Kit (Vazyme, #RC401). RNA integrity was verified by 1.2% agarose gel electrophoresis using a Bio-Rad Gel Doc XR+ system, while concentration and purity (A260/A280 ratio ≥1.8) were measured with a NanoDrop One spectrophotometer (Thermo Fisher Scientific). First-strand cDNA was synthesized from 1 μg total RNA using HiScript II QRT SuperMix for qPCR (Vazyme, #R223) according to the manufacturer’s protocol.

### 2.3 Primer design and vector construction

The hexaploid Camellia oleifera ‘ASUS’ genomic data used in this study was independently sequenced by our research group (unpublished), and the sequencing was independently completed by our research group. The transcriptome data were derived from the kernel tissue in the early stage of lipid synthesis. Original data can be obtained by contacting the corresponding author (email: jiangyaocn@126.com). The gene amplification primers were designed according to the sequence, and the gene amplification primers were designed by online software (Primer3Plus-Pick Primers). The gene fragment was inserted into the vector *pCAMBIA1300* by seamless cloning using the kit ColnExpress^®^IOne Step Cloning Kit (Vazyme, #C112).

### 2.4 Cloning and identification of *CoTTG1*

The complete coding frame fragment of the gene was obtained by PCR using the seed cDNA of ‘Asus’ as the material. Subcellular localization prediction was performed using PSORT Prediction Tool (https://psort.hgc.jp/form2.html). The transmembrane structure was analyzed by online software TMHMM Server v2.0 (http://www.cbs.dtu.dk/services/TMHMM). The signal peptide was predicted by the online software SignalP-5.0 (http://www.cbs.dtu.dk/services/SignalP/). GSDS (http://gsds.cbi.pku.edu.cn) was used to analyze the intron and exon structure of the gene. The motif of the protein was searched by MEGA 11 (http://meme-suite.org). Homologous amino acid sequences were retrieved by NCBI (https://www.ncbi.nlm.nih.gov), and sequence alignment was performed by SnapGene software. The phylogenetic tree of homologous proteins was constructed by MEGA software, and the neighbor-joining (NJ) method was used. The number of self-help repetitions was set to 1000, and other parameters were default.

### 2.5 Real-time quantitative polymerase chain reaction

The expression of genes in the samples was detected by real-time quantitative polymerase chain reaction (RT-qPCR) using different tissues of *C. oleifera* seeds and transgenic *Arabidopsis* plants at different developmental stages. Each treatment included three biological replicates and three technical replicates. RT-qPCR was performed according to the instructions of Taq Pro Universal SYBR qPCR Master Mix (Vazyme), and CFX96 Real-Time PCR System was used for detection. The relative gene expression level was quantified using *GAPDH* as an internal reference gene (Meng et al., 2018) and calculated using the 2^−ΔΔCt^ method (Schmittgen, 2008).

### 2.6 Agrobacterium-mediated Transformation and Transgenic Screening of *Arabidopsis thaliana*

*Arabidopsis thaliana* transformation was performed using the floral dip method (Zhang et al., 2006). Briefly, Agrobacterium tumefaciens strain GV3101 harboring the binary vector was grown in LB medium with appropriate antibiotics to OD_600_ = 1.5–2.0. Bacterial cells were pelleted and resuspended in 5% (w/v) sucrose solution containing 0.02% (v/v) Silwet L-77. Primary inflorescences of 4-week-old A. thaliana (ecotype Col-0) were dipped in the suspension for 10 s, then covered with plastic wrap and maintained at high humidity for 24 h. Plants were grown to maturity under long-day conditions (16-h light/8-h dark) until seed harvest. For transgenic screening, T1 generation seeds were surface-sterilized with 70% ethanol (1 min) followed by 50% bleach/0.05% Tween-20 (10 min), rinsed thrice with sterile water, and suspended in 0.05% agarose. Seeds were plated on ½MS medium (4.3 g/L Murashige-Skoog salts, 10 g/L sucrose, 0.5 g/L MES, 8 g/L agar, pH 5.7) supplemented with 25 mg/L hygromycin and 100 mg/L carbenicillin. After stratification at 4°C for 3 d, plates were transferred to a growth chamber (22°C, continuous light). Putative transformants were identified 7–10 d post-germination by vigorous root growth and green true leaves, while non-transformants showed stunted roots. Resistant seedlings were transplanted to soil for further analysis.

### 2.7 Subcellular localization of *CoTTG1* protein

The pCAMBIA1300-*CoTTG1*:GFP recombinant vector was constructed and transformed into *Agrobacterium tumefaciens* GV3101.The positive Agrobacterium was inoculated into LB liquid medium (containing 50 μg/mL kanamycin, 50 ug/mL rifampicin), 28°C, 200 rpm constant temperature shaking culture overnight. The cultured bacterial solution was centrifuged in a centrifuge at 5000 rpm for 8 minutes, and the supernatant was discarded and added to the buffer (10 mM MES, pH 5.6, 10 mM MgCl_2_, 200 μM acetosyringone).The bacterial solution was resuspended, and the OD600 value of the bacterial solution was adjusted to 0.8 and incubated at room temperature for 2 h. *Nicotiana benthamiana* was used as the material. Agrobacterium containing *35s: CoTTG1-GFP* expression vector and Agrobacterium containing nuclear localization NLS-mcherry red fluorescent protein were mixed in equal volume and infiltrated into the abaxial leaf surface using a needleless syringe of tobacco leaves. After 24 hours of dark culture at room temperature and 24 hours of low light culture, we collected samples. Confocal laser scanning microscopy (Zeiss LSM 700; excitation/emission wavelengths: 488 nm/510–550 nm for GFP, 587 nm/610 nm for mCherry) was used to observe and collect images.

### 2.8 Analysis of trichomes

Trichome density was quantified on the fifth rosette leaf of *Arabidopsis thaliana* using a Leica MS5 dissecting microscope (Leica Microsystems) at 20×magnification. For each genotype (e.g., WT and transgenic lines), ten plants were analyzed with three biological replicates (independent growth cycles). Only trichomes protruding clearly from the adaxial leaf surface within a defined 1-mm² mid-lamina region were counted. Trichome clustering patterns were classified according to established criteria (Larkin, 1994).

### 2.9 Microscopic observation and histochemistry analysis

Determination of proanthocyanidins: first, *Arabidopsis* seeds were placed in a dark environment at room temperature. Subsequently, the seeds were stained with dimethyl cinnamaldehyde (DMACA) reagent, 0.5 g DMACA powder was dissolved in 100 ml solution (50 ml 3 mol/L hydrochloric acid solution and 50 ml 50% methanol solution), and then the pretreated seeds were added to 100 ml dyeing solution. The dyeing process lasted for 48 hours. After dyeing, the seeds were washed three times with 70% ethanol to remove excess dye. The treated seeds were stained with Nikon SMZ1500 (Nikon) or Axio Imager 2 (Carl Zeiss).

### 2.10 Determination of anthocyanin content

Take 0.1 g of the young leaves of the plant to a 1.5 mL centrifuge tube, and then quickly placed in liquid nitrogen for freezing and fully ground. Add 0.6ml 1% (v/v) HCl in methanol, soak overnight at 4°C. Then 0.4 mL water and 0.4 mL chloroform were added to the sample. Fully mixed, centrifuged at 12,000 rpm for 5 min at room temperature. Transfer the supernatant to a new 1.5 mL centrifuge tube. The content of anthocyanin in the supernatant was detected by microplate reader, and the detection wavelengths were 530 nm and 657 nm. The anthocyanin content in the leaves was calculated according to the obtained data. The calculation method is as follows: anthocyanin content*(*mg/g*)*=*(*OD_530_-0.25×OD_657_ *)*/M, M is the weight of the material.

### 2.11 Determination of oil content and fatty acid composition by gas chromatography

The oil content of *C. oleifera* seeds was determined by Soxhlet extraction method. The fresh seeds of different periods and treatments were dried to constant weight at 60°C, and then grinded into powder using a grinder (FW-80). About 5.0 g seed powder (recording mass m1) was weighed and put into a filter paper tube, sealed at both ends and tied with a thin line, and then put into a soxhlet extractor. The mass of the aluminum oil cup before extraction was weighed as M1, and about 50ml petroleum ether was added and connected to the Soxhlet extractor (Hanon SOX406). The extraction temperature was set to 75°C.The extraction process includes: boiling in the solvent with a tube for 30 minutes, rinsing for 150 minutes, and evaporating for 60 minutes to recover the solvent. After extraction, the mass of the aluminum oil cup was weighed as M2, and the extracted oil was poured out and stored in a centrifuge tube. Each treatment was repeated three times, and the oil content calculation formula was as follows: oil condition content (%) = (M2-M1)/m1×100%.

The oil content of *Arabidopsis thaliana* seeds was determined by micro-extraction method using a gas chromatograph (Shimadzu GC-2014, China Shimadzu Enterprise Management Co., Ltd.). The *Arabidopsis thaliana* seeds were dried in an oven at 60°Cto constant weight and crushed with a high-speed grinder. Micro-weighed powder R1 = 100 mg, placed in a 15 mL sterile centrifuge tube, added 4 mL n-heptane, 100 μL C12:0 internal standard, and intermittently oscillated using a vortex oscillator at room temperature for 5 min. Adding 200 μL 2 mol/L potassium hydroxide methanol solution, the preparation method of potassium hydroxide methanol solution is as follows: 1.31 g potassium hydroxide was dissolved in 10 mL anhydrous methanol, heated slightly, added anhydrous sodium sulfate to dry, filtered, oscillated for 30 s, until the solution was clear. The supernatant was drawn using a 1 mL syringe, filtered using a 0.45 μm organic needle filter, and the filtrate was transferred to the injection bottle. The fatty acid composition of *Arabidopsis thaliana* seeds was determined by the internal standard method in ‘Determination of fatty acids in food safety national standard food oil condition content: w% = (4/e-4)/R1 (e is the percentage of internal standard.).

The parameters of gas chromatograph are as follows: FID detector temperature is 250°C, inlet temperature is 250°C.The chromatographic conditions were as follows:flame ionization detector, chromatographic column (60 m×0.25 mm×0.2 μm), carrier gas of nitrogen, split ratio of 1:50, automatic injection of 1 μL. The heating procedure was set as follows: first, the temperature was maintained at 50°C for 2 minutes, then the temperature was increased to 170°C at 10°C/min and maintained for 10 minutes, then the temperature was increased to 180°Cat 2°C/min and maintained for 10 minutes, and finally the temperature was increased to 220°C at 4°C/min and maintained for 22 minutes.

## 3 Results

### 3.1 Cloning and identification of *CoTTG1* gene

**Figure 1:**
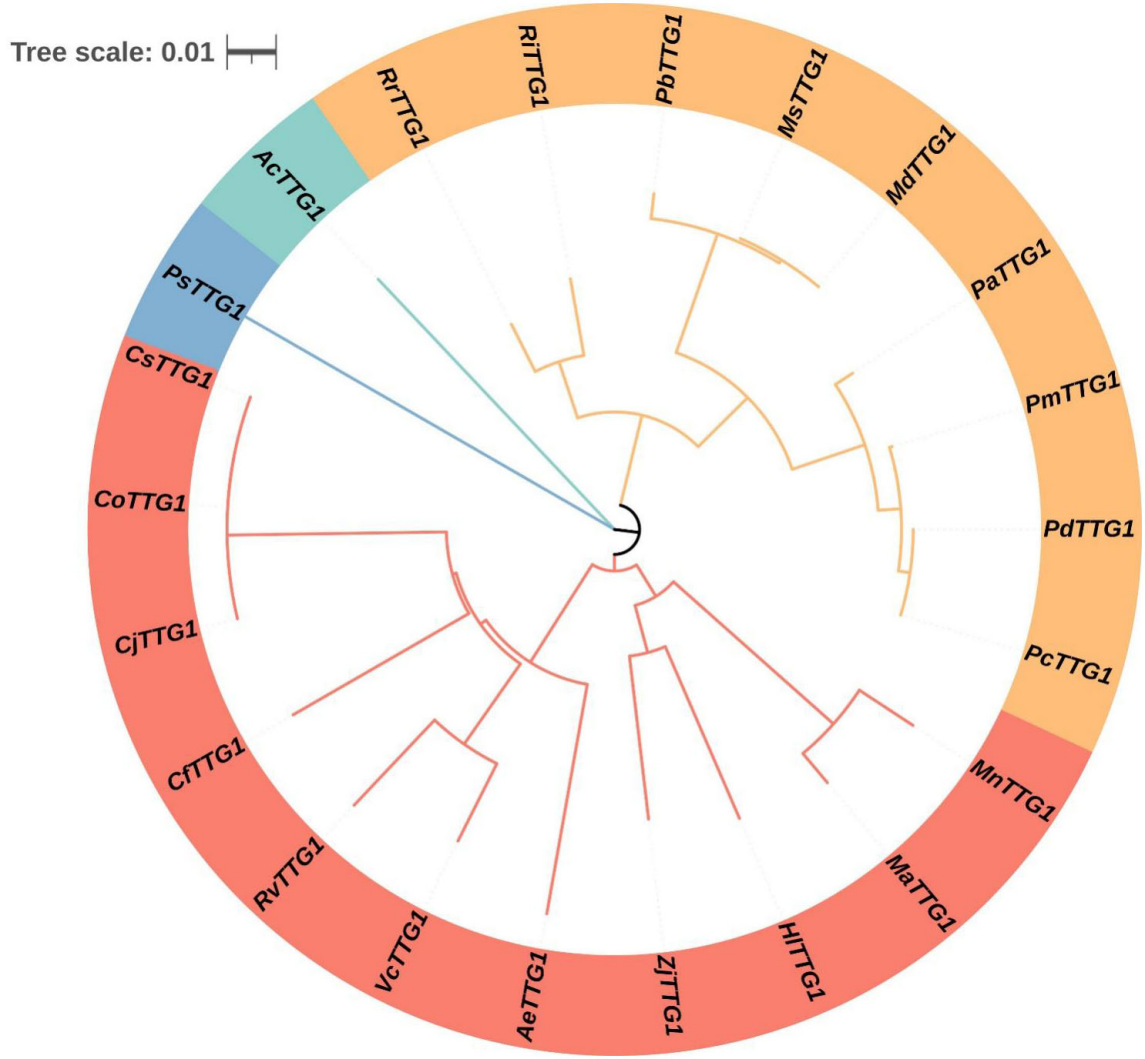
Phylogenetic tree analysis of *CoTTG1* in *Camellia oleifera*. Cj, *Camellia japonica*; Cs, *Camellia sinensis*; Ae, *Actinidia eriantha*; Cf, *Cornus florida*; Rv, *Rhododendron vialii*; Vc, *Vaccinium corymbosum*; Zj, *Ziziphus jujuba*; Pa, *Prunus avium*; Pp, *Prunus persica*; Pc, *Prunus armeniaca cultivar Chuanzhihong*; Md, *Malus domestica*; Pm, *Prunus mume*; Ms, *Malus sylvestris*; Pd, *Prunus dulcis*; Ri, *Rubus idaeus*; Hl, *Humulus lupulus*; Ma, *Morus alba*; Rr, *Rosa rugosa*; Mn, *Morus notabilis*; Pb, *Pyrus betulifolia*; Ac, *Allium cepa*; Ps, *Pisum sativum*; Mn, *Musa nanensis*; Pd, *Phoenix dactylifera*.

The gene fragment with a length of 1100 bp was obtained by PCR. The gene sequencing results showed that the amino acid sequence of the gene had a high sequence similarity with the *TTG1* amino acid sequence of multiple species, indicating that the *TTG1* gene of *C. oleifera* was successfully cloned. In order to fully understand the evolutionary relationship and homology of *CoTTG1* with *TTG1* of other species, the amino acid sequence of *CoTTG1* was similar to that of *TTG1* protein of other species. Due to the close evolutionary relationship between *C. oleifera* and *Camellia japonica*, the results showed that the similarity between *C. oleifera* and *C. japonica* was as high as 99.71%. Secondly, the similarity between *C. oleifera TTG1* and *Actinidia eriantha* (kiwifruit) reached 86.1%, and the similarity between *C. oleifera TTG1* and *Taxus orientalis* reached 86%, and the similarity between *C. oleifera TTG1* and *Rhododendron simsii*, *sorghum blueberry* reached 84%, and the similarity between *C. oleifera TTG1* and *jujube* reached82%. In addition, the similarity between *C. oleifera TTG1* and *cherry*, *peach*, *apricot* reached 81% (**Figure 1**). The high sequence similarity reflects the evolutionary conservation and functional importance of *TTG1*. In addition, the *TTG1* gene can be clearly divided into three main evolutionary branches (or called evolutionary clusters). The *TTG1* gene of *C. oleifera* and the *TTG1* gene of *C. japonica* were closely clustered in the same branch on the evolutionary tree. This finding suggests that there is a very close genetic relationship and evolutionary relationship between the *TTG1* genes of *C. oleifera* and *C. japonica*.

The results of protein physical and chemical analysis showed that the molecular weight of *CoTTG1* protein was 38.3781 KDa, the molecular formula was C_1695_H_2617_N_463_O_528_S_14_, and its theoretical isoelectric point (pI) was 5. *CoTTG1* contains 45 negatively charged residues, mainly composed of aspartic acid (Asp) and glutamic acid (GLu), and 28 positively charged residues, composed of arginine (Arg) and lysine (Lys).The instability index (II) of the protein was 55.88, which was an unstable protein. Hydrophobicity analysis revealed the hydrophilic nature of *CoTTG1*, indicating that it is more likely to interact with water molecules than with lipid membranes. No obvious signal peptide sequence was detected in the sequence of *CoTTG1*.

### 3.2 Expression results and analysis of *TTG1* gene in *Camellia oleifera*

**Figure 2:**
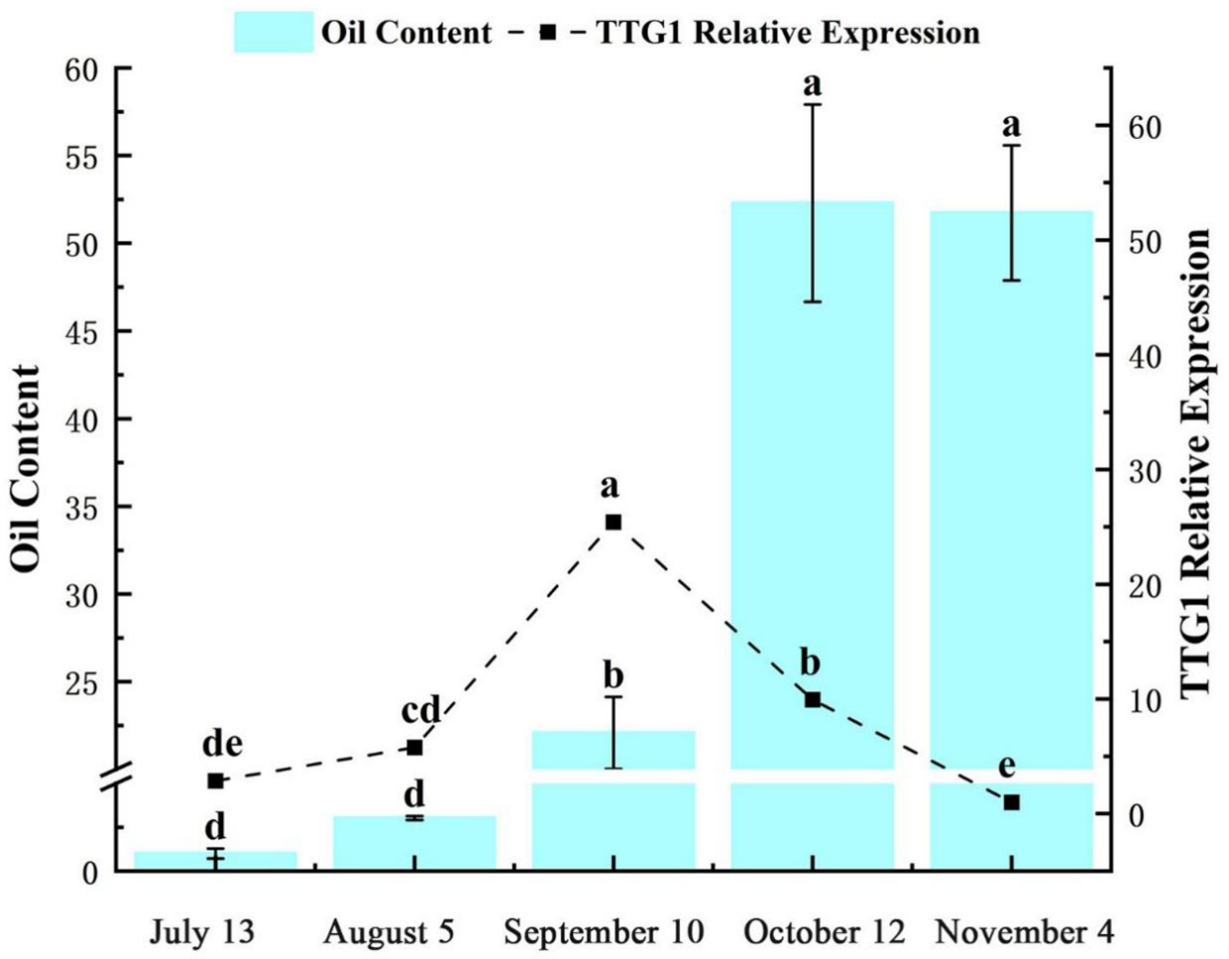
Oil content and relative expression of *CoTTG1* in *Camellia oleifera* seeds at different developmental stages. Letters indicate a significant difference at the p < 0.05 level.

The expression level of *TTG1* gene in *C. oleifera* seed shows a specific dynamic change rule with time. From July 13 (globular embryo to heart-shaped embryo formation stage, the seed was in the filling stage of colloidal substances, and the oil content was close to zero), the expression level of *TTG1* gene gradually increased. On September 10 (cotyledon embryo maturity stage, the seed was full and the number of oil bodies increased significantly, and the oil accumulation was rapidly initiated), the expression level of this gene continued to decrease (**Figure 2**). July 13th, the seed oil content of *C. oleifera* approached zero. As the fruit develops to August 5th (the transition period from torpedo-shaped embryo to cotyledon-shaped embryo, the endosperm disintegrates and the seed turns milky white) (Verma et al., 2022), the oil content begins to increase slowly; From September 10th to October 12th (from the maturity of cotyledonous embryos to the early stage of fruit maturity, with the oil body accounting for 1/3 of the cell volume), the oil content of the seed seeds increased significantly; From October 12th to November 4th (the period when the fruit is fully ripe, the seed coat hardens and the oil accumulation is complete), the oil content enters the plateau period (**Figure 2**). This indicates that from September 10th to October 12th is precisely the critical period for the rapid accumulation of seed oil. Correlation analysis showed that the trends of oil synthesis and accumulation in *C. oleifera* seeds and the expression characteristics of the *TTG1* gene were similar, and the peak of gene expression was earlier than the peak of oil accumulation (for example, the peak of gene expression on September 10th corresponds to the acceleration of oil accumulation on October 12th). Combined with the development process of *C. oleifera* seeds, it is speculated that the *TTG1* gene may play a regulatory role in the upstream of the oil synthesis pathway-especially at the mature stage of cotyledonous embryos (September 10th), it activates the key genes involved in oil body formation or fatty acid metabolism through transcription, thereby driving the subsequent rapid accumulation of oil.

### 3.3 Subcellular localization of *CoTTG1*

**Figure 3:**
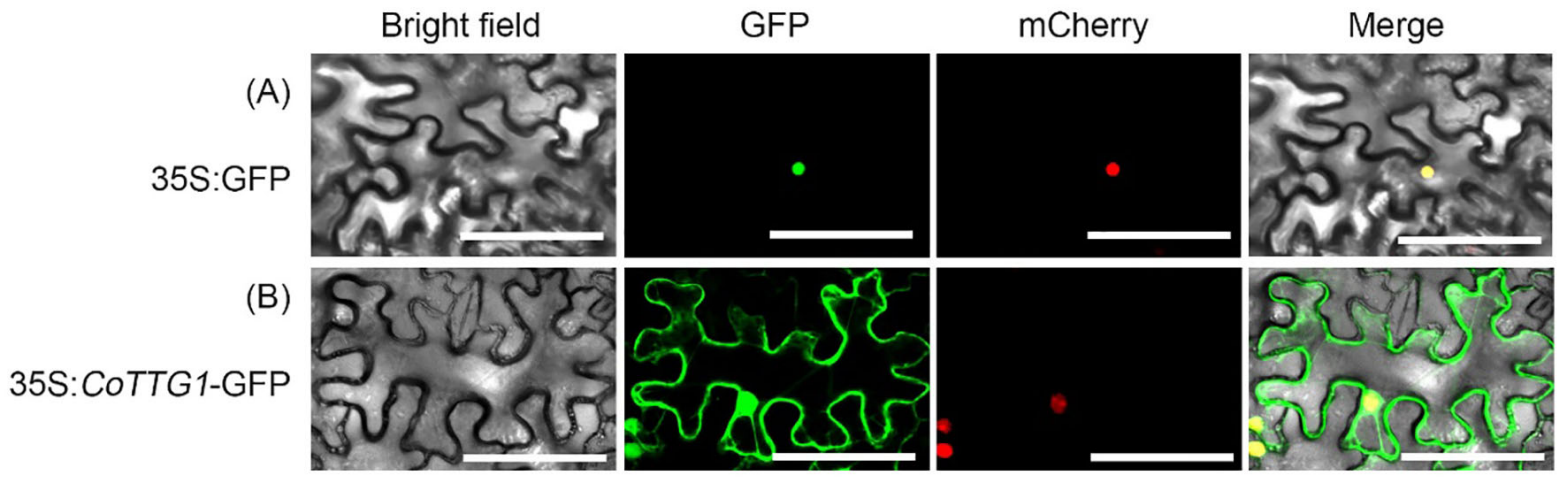
Observation map of subcellular localization of *CoTTG1*. **(A)** 35S:GFP **(B)** 35S:CoTTG1-GFP Bar = 200 µm.

The green fluorescence signal of *CoTTG1* overlapped with the red fluorescence signal of nuclear localization marker (**Figure 3**), and the expression signal was clear and strong, indicating that *CoTTG1* was localized in the nucleus, which was consistent with the prediction results.

### 3.4 Expression analysis of *CoTTG1* in transgenic plants

**Figure 4:**
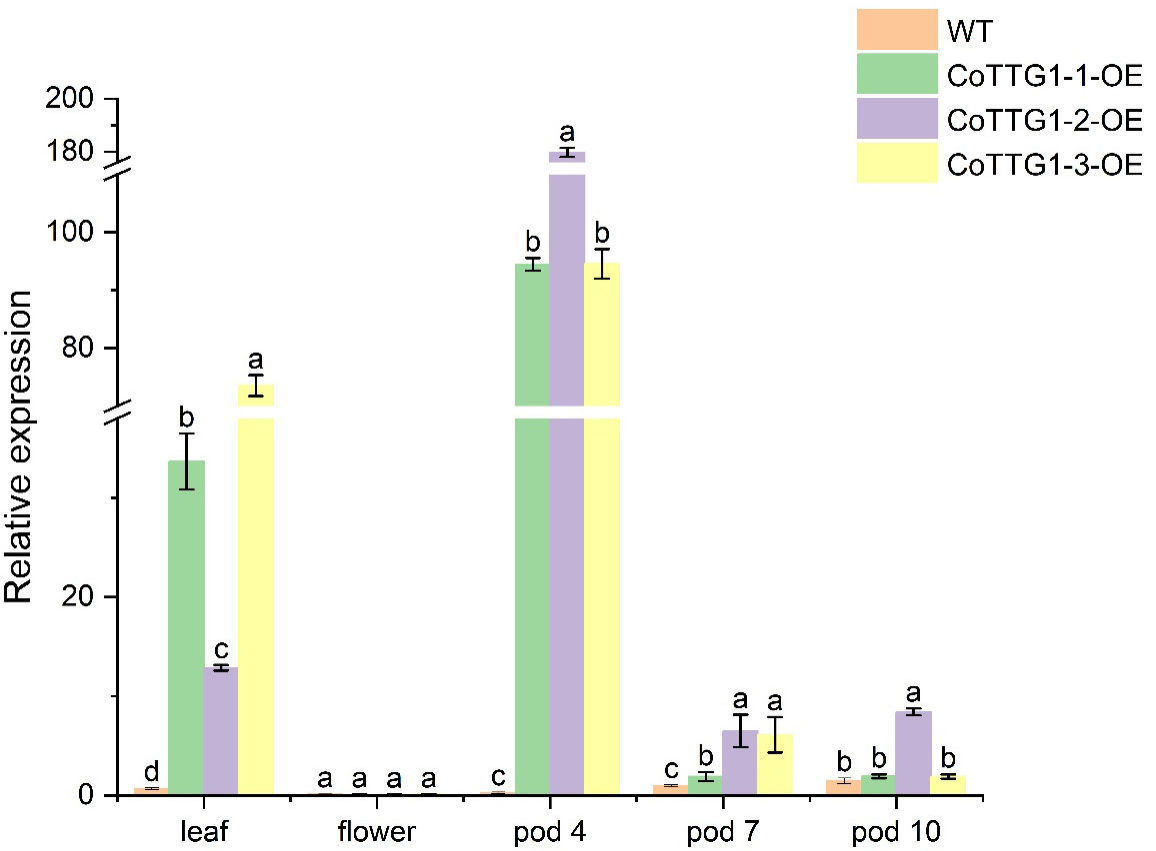
Relative expression analysis of *CoTTG1* gene in overexpression transgenic *Arabidopsis thaliana*. Letters indicate a significant difference at the p<0.05 level.

In order to further study the role of *CoTTG1* gene in the development of embryo development and anthocyanins, the expression levels of *CoTTG1* gene in *Arabidopsis* leaves, flowers and seeds at 10d, 12d and 14d after flowering (represented as pods 4,7 and 10, At these stages, the embryos within the seeds are in different maturation phases, for example, 10 days corresponds to the heart-shaped to torpedo-shaped embryo stage, 12 days corresponds to the curved cotyledon stage, and 14 days corresponds to the mature embryo stagey) were detected by RT-qPCR (**Figure 4**). The results showed that the expression level of *CoTTG1* in overexpression plants was increased in leaves and seeds, and the expression level was 51.6 times and 0.8 times higher than that of wild type, respectively (**Figure 4**). The expression of *CoTTG1* gene at the developmental stage of *Arabidopsis thaliana* showed that the expression levels of *CoTTG1*-1, *CoTTG1–*2 and *CoTTG1–*3 in transgenic lines were significantly higher than those in wild type at the 4th stage of pod (green young fruit stage, corresponding to the heart-shaped to torpedo-shaped embryo stage). The expression levels were 356 times, 678 times and 356 times, respectively. The expression level of *CoTTG1* transgenic lines was 1.9-6.5 times higher than that of wild type in pod stage 7) middle development stage, corresponding to the curved cotyledon stage). At the pod 10 stage (pre-maturation stage, corresponding to the mature embryo stage), the expression level of *CoTTG1* transgenic lines was 1.3-5.8 times that of WT (**Figure 4**). With the gradual ripening of the fruit, the relative expression of *CoTTG1* showed a downward trend from the pod 7 stage to the pod 10 stage (**Figure 4**).

### 3.5 Overexpression of *CoTTG1* in *Arabidopsis* can promote the formation of trichomes.

**Figure 5:**
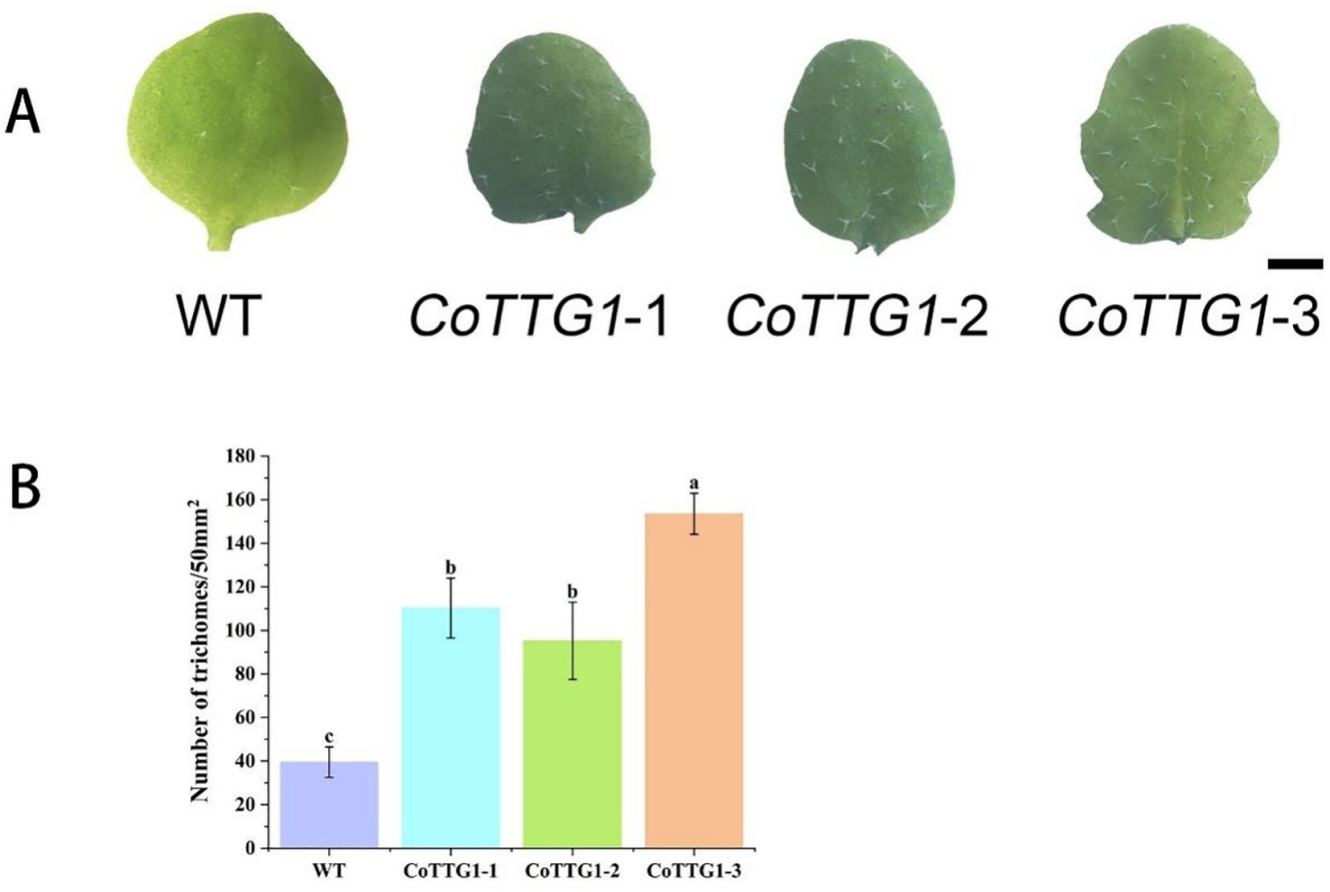
Quantitative analysis of trichomes in leaves of overexpressing *CoTTG1* plants. **(A)** Wild type (WT) *Arabidopsis* and overexpression lines of the fifth true leaf trichomes (bar = 200μm); **(B)** The number of trichomes per 50mm2 area of the 5th true leaf of WT and overexpression lines was counted, *P < 0.05. Letters indicate a significant difference at the p<0.05 level.

The overexpression of *CoTTG1* gene in *Arabidopsis* significantly increased the number of trichomes in leaves. Compared with wild-type *Arabidopsis* plants, the number of trichomes on the leaves of the three overexpression lines of *CoTTG1*-1, *CoTTG1–*2 and *CoTTG1–*3 increased significantly (**Figure 5**). The statistical results of the fifth leaf showed that the average number of trichomes on the 50 mm^2^ leaf area of the overexpression lines increased by 80. Among them, the *CoTTG1–*3 strain was the most prominent, and the average number of trichomes on its leaves increased by 114 (**Figure 5**); the *CoTTG1–*1 and *CoTTG1–*2 lines increased about 71 and 56 trichomes, respectively (**Figure 5**). These results indicate that the *TTG1* gene of *C. oleifera* may play an important role in the regulation of leaf trichome formation.

### 3.6 Expression analysis of epidermal hair related genes in transgenic plants

**Figure 6:**
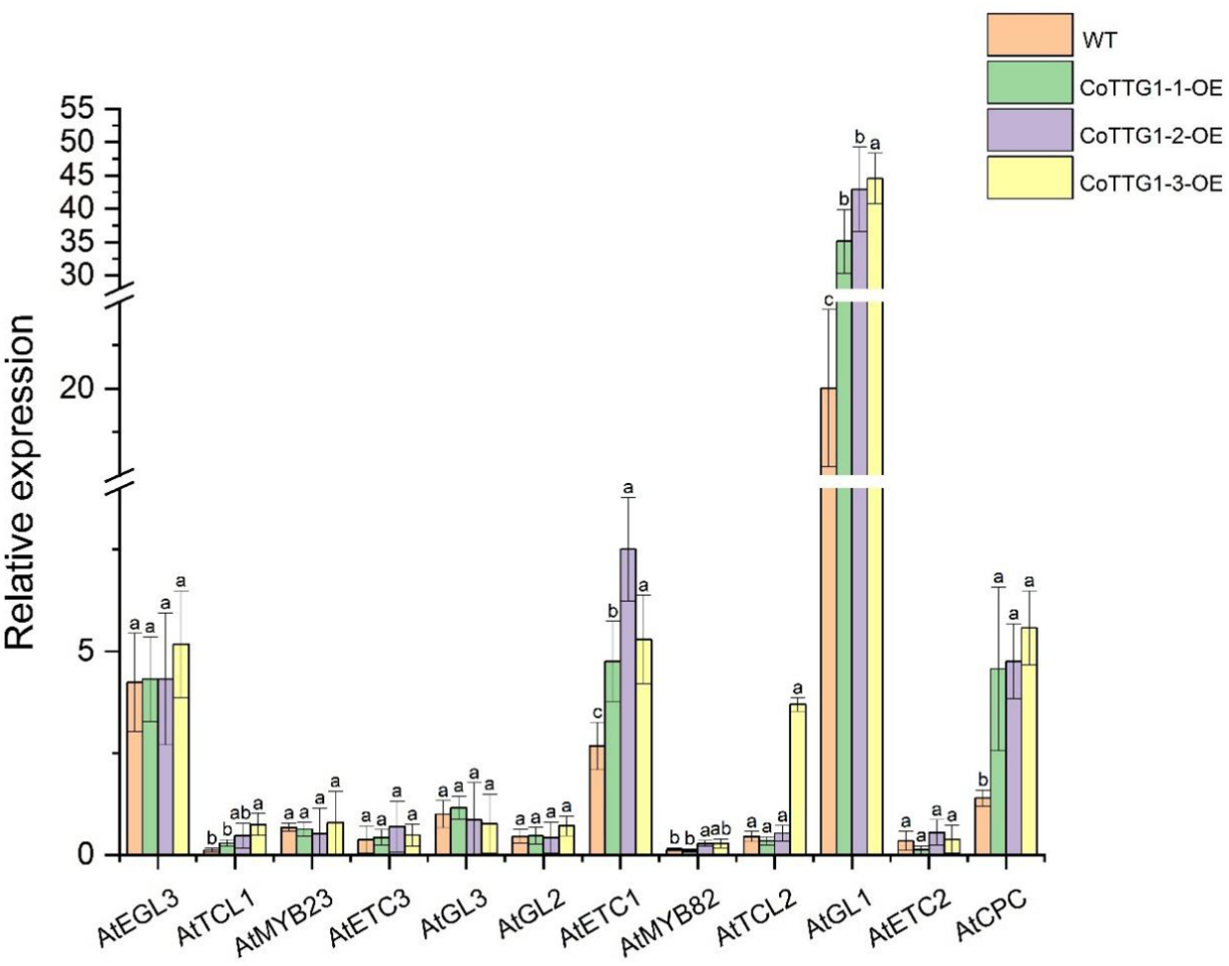
Analysis of expression levels of genes related to trichome formation in overexpressing transgenic plants. Letters indicate a significant difference at the p<0.05 level.

**Figure 7:**
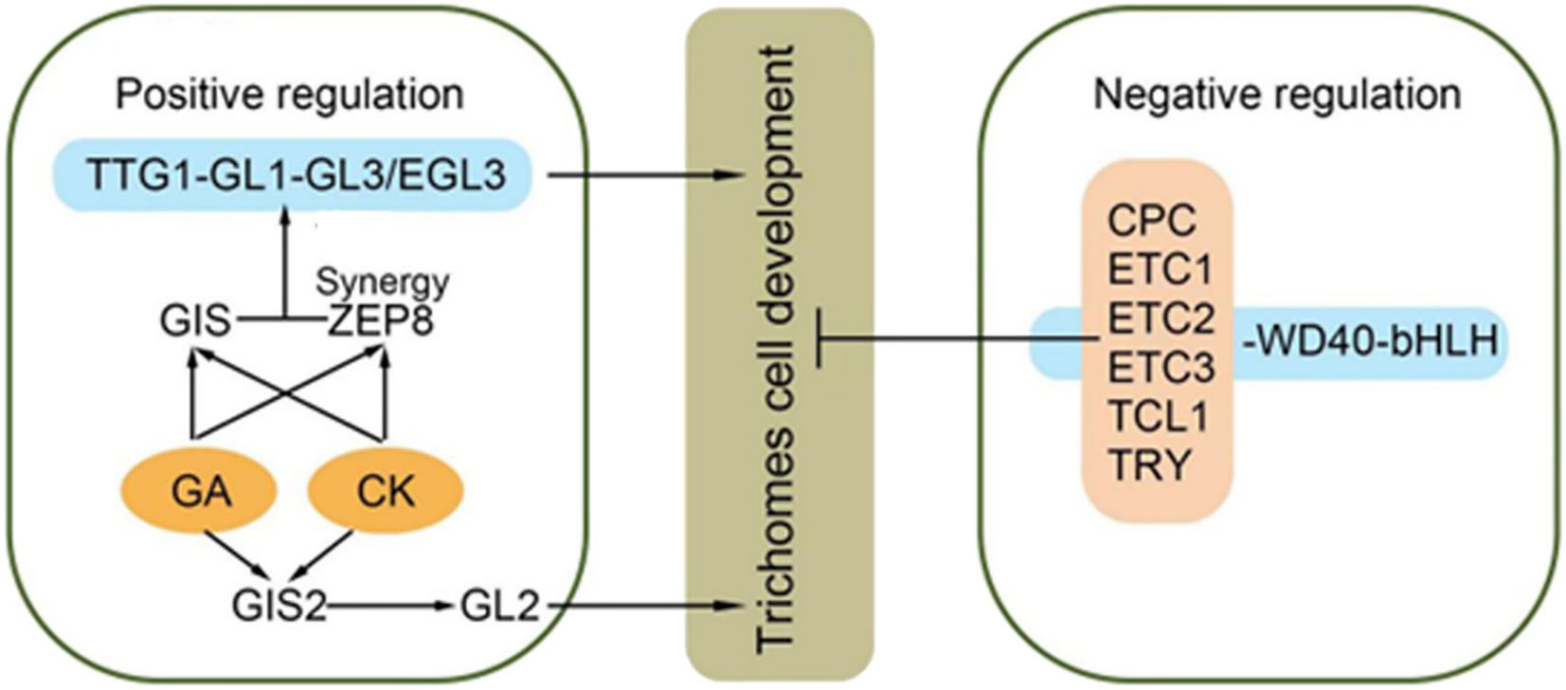
Epidermal hair synthesis pathway [Adapted from LIU et al (Liu et al., 2021)]:*GLABRAI (GLI)、GLABRA3 (GL3)、GLABRA 2 (GL2)、ENHANCER OF GLABRA3 (EGL3)、CAPRICE (CPC)、TRIPTYCHON (TRY)、ENHANCEROFTRYANDCPCI&2&3 (ETCI&2&3)、MYB MIXTA-like 8*.

In the transgenic plants overexpressing *CoTTG1*, the expression levels of three genes *AtETC1*, *AtGL1* and *AtCPC* closely related to trichome development showed a significant increase, while the expression levels of other genes related to trichome development, such as *AtETC3*, *AtTCL1*, *AtTCL2*, *AtETC2*, *AtMYB82*, *AtGL3*, *AtMYB23* and *AtGL2*, did not change significantly (**Figure 6**). *CoTTG1* gene may effectively promote the formation and development of *Arabidopsis* trichomes by enhancing the expression levels of *AtETC1*, *AtGL1* and *AtCPC*. In the transgenic plants overexpressing *CoTTG1*, despite elevated expression of trichome inhibitors *AtETC1* and *AtCPC*, we observed a significant increase in trichome density. This apparent contradiction can be explained through coordinated regulatory mechanisms: (1) The dramatic upregulation of *AtGL1* - a core MYB transcription factor that forms an activation complex with *TTG1* and *GL3*/*EGL3* - provides strong developmental impetus; (2) The activation signal from *AtGL1* exceeds the inhibition threshold, creating a dose-dependent advantage; and (3) *TTG1* may modulate *AtETC1*/*CPC* activity through protein interactions, potentially altering their subcellular localization or function. The concurrent upregulation of both activators and inhibitors likely represents an autonomous feedback mechanism that permits increased trichome formation while preventing excessive proliferation. These results indicate that the formation of trichomes depends on the dynamic balance of positive and negative regulatory factors, and the overexpression of *CoTTG1* ultimately promotes the development of trichomes by enhancing the core positive regulatory network (**Figure 7**). The regulatory network involves GL1-GL3-TTG1 interactions, while increased expression of inhibitors like CPC may provide feedback regulation.

### 3.7 *CoTTG1* positively regulates anthocyanin synthesis

**Figure 8:**
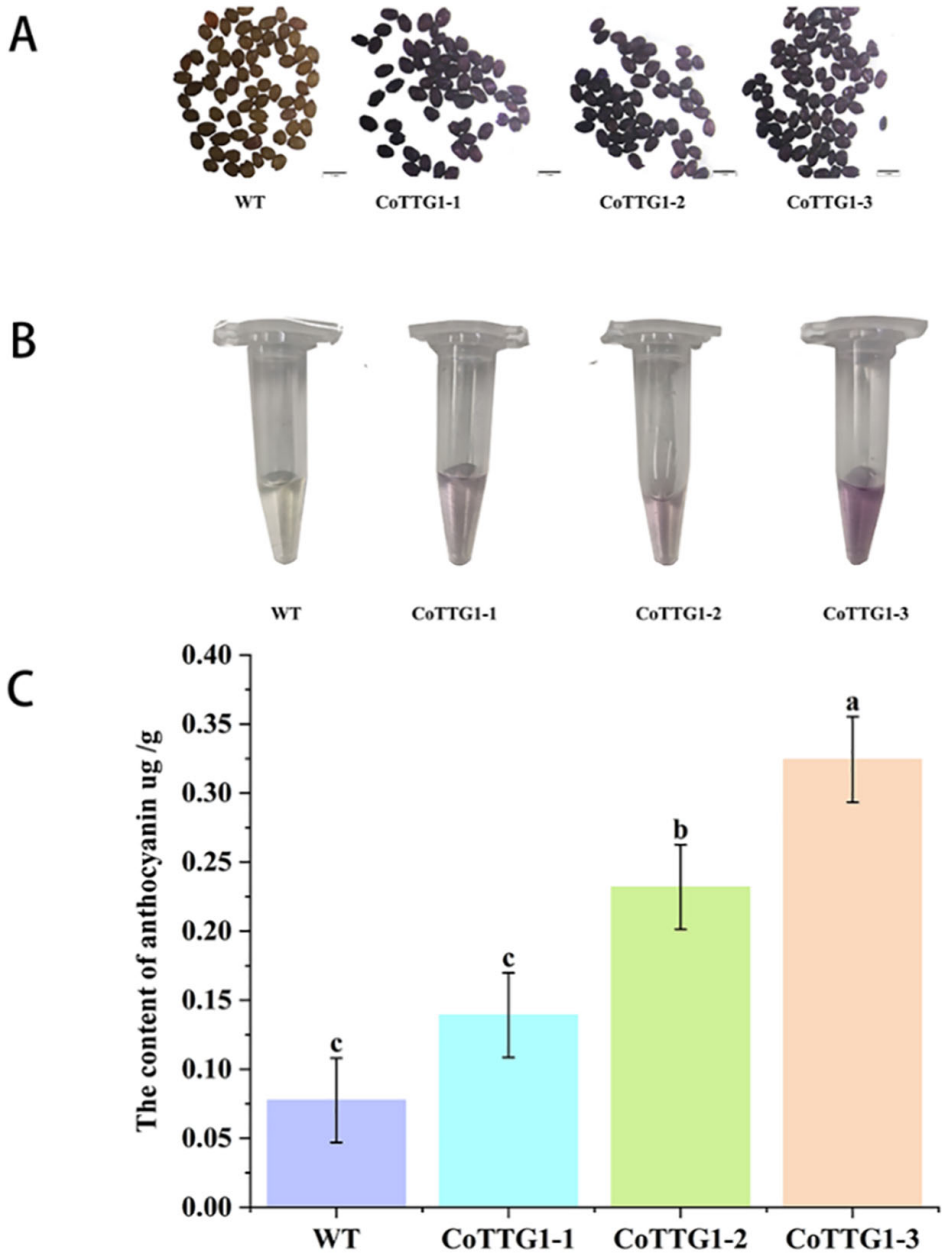
The results of DMACA staining and anthocyanin content in leaves of overexpressing *CoTTG1* plants. **(A)** Comparison of seed staining between wild type (WT) *Arabidopsis* and overexpression lines. **(B)** Color comparison of leaf extract between WT and overexpression lines. **(C)** The anthocyanin content in the leaves of WT and overexpression lines was compared, *P < 0.05. Letters indicate a significant difference at the p<0.05 level.

It was found that the seed coat color of mature seeds of *CoTTG1*-1, *CoTTG1–*2 and *CoTTG1–*3 overexpressing *Arabidopsis* lines was significantly darker than that of wild type (**Figure 8**). The results of DMACA staining showed that the overexpression lines were stained deeper. Heterologous overexpression of *CoTTG1* in *Arabidopsis thaliana* may deepen the color of seeds by promoting the synthesis of proanthocyanidins (**Figure 8**). The seed coat color of CoTTG1 overexpression lines was significantly darker than wild type (**Figure 8A**), and DMACA staining confirmed deeper pigmentation. Additionally, spectrophotometric analysis revealed a 199–318% increase in anthocyanin content in leaves (**Figure 8C**). The results of microplate reader showed that the anthocyanin content of *CoTTG1* overexpression lines increased by 199%-318% compared with the wild type, which was consistent with the phenotype observed in the leaf extract in **Figure 8B** (**Figure 8**). This result demonstrates that *CoTTG1* gene may play an important role in positively regulating anthocyanin accumulation.

### 3.8 Analysis of anthocyanin-related gene expression in transgenic plants

**Figure 9:**
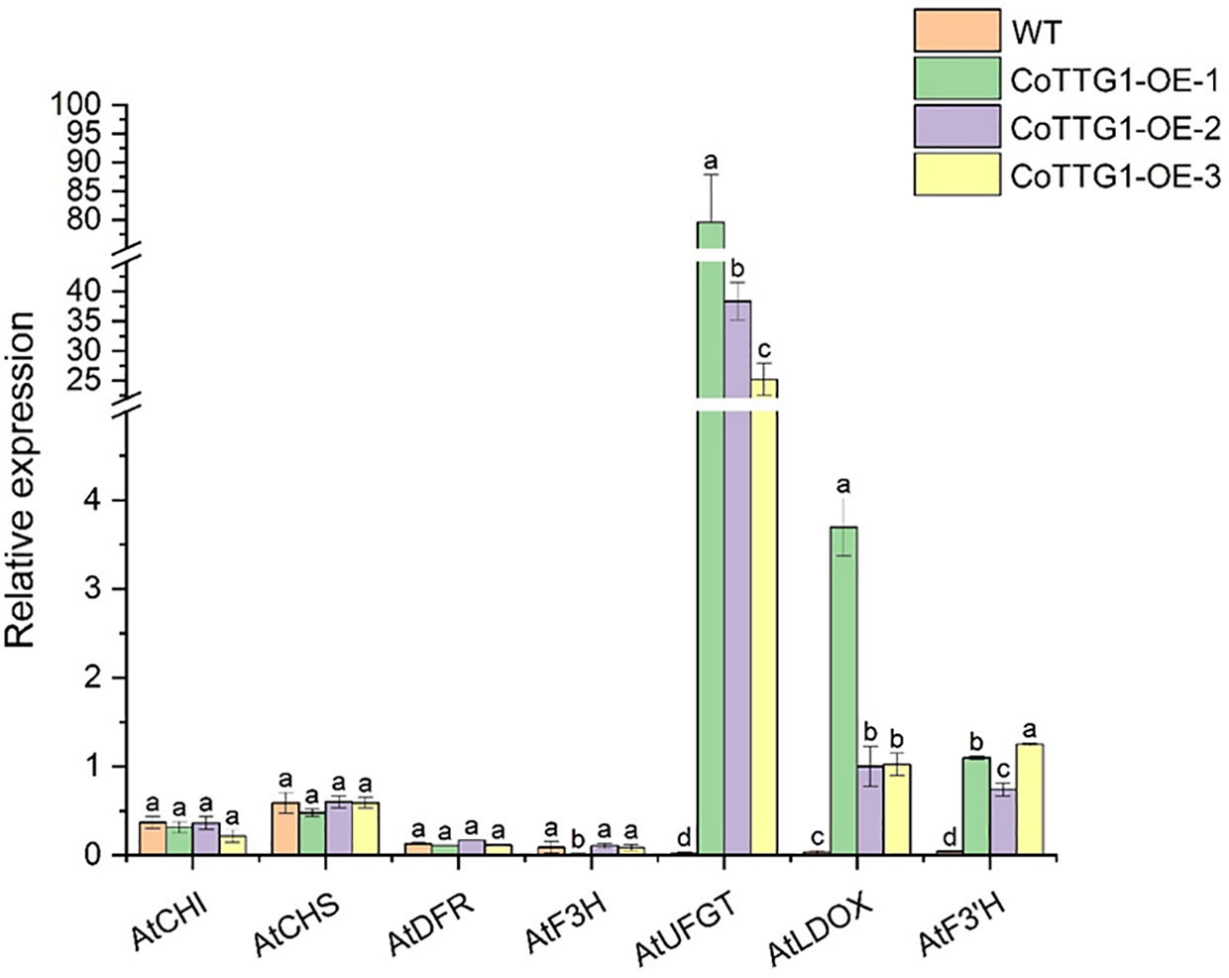
Quantitative analysis of the expression levels of genes related to anthocyanin synthesis in the leaves of transgenic plants. Letters indicate a significant difference at the p<0.05 level.

**Figure 10:**
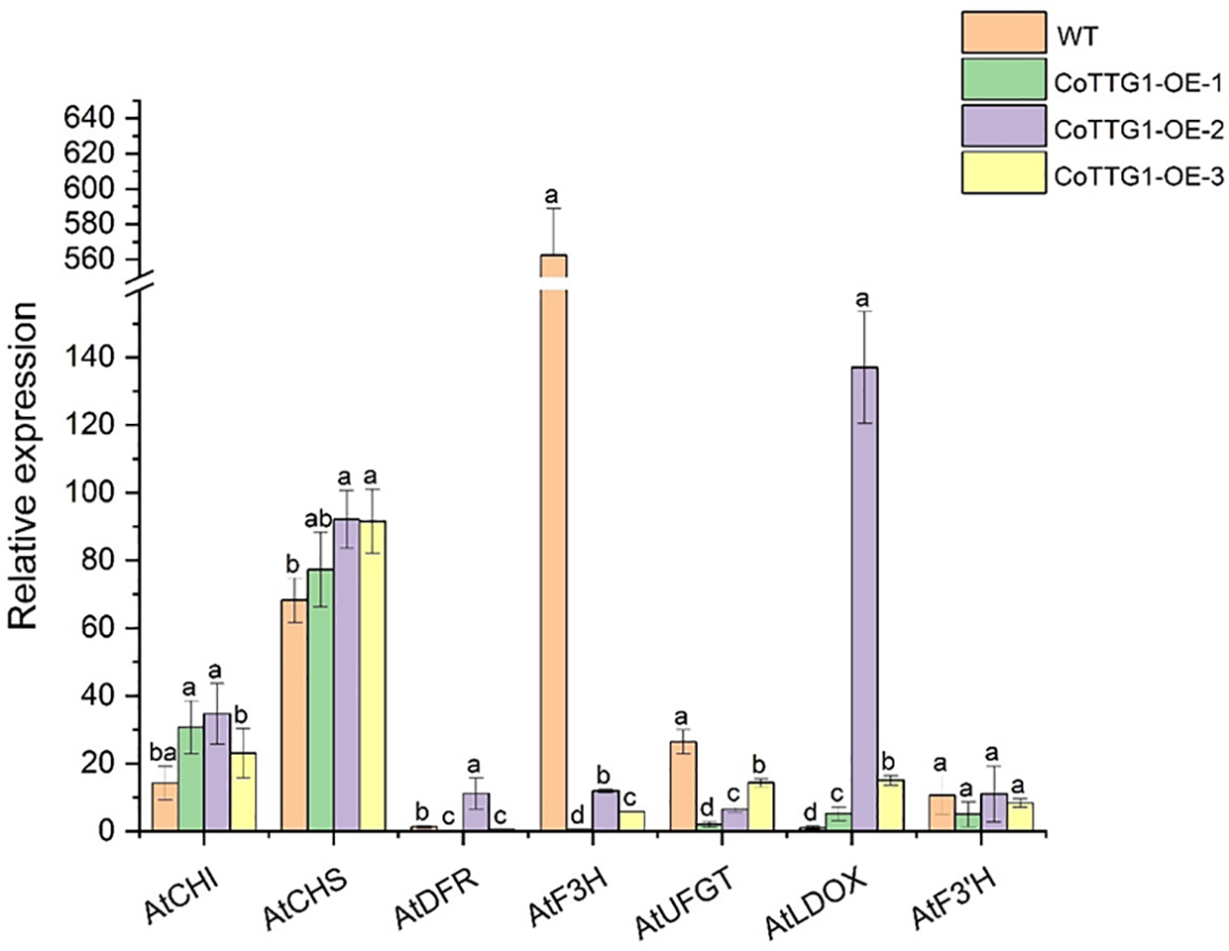
Quantitative analysis of the expression levels of genes related to anthocyanin synthesis in flowers of transgenic plants. Letters indicate a significant difference at the p<0.05 level.

**Figure 11:**
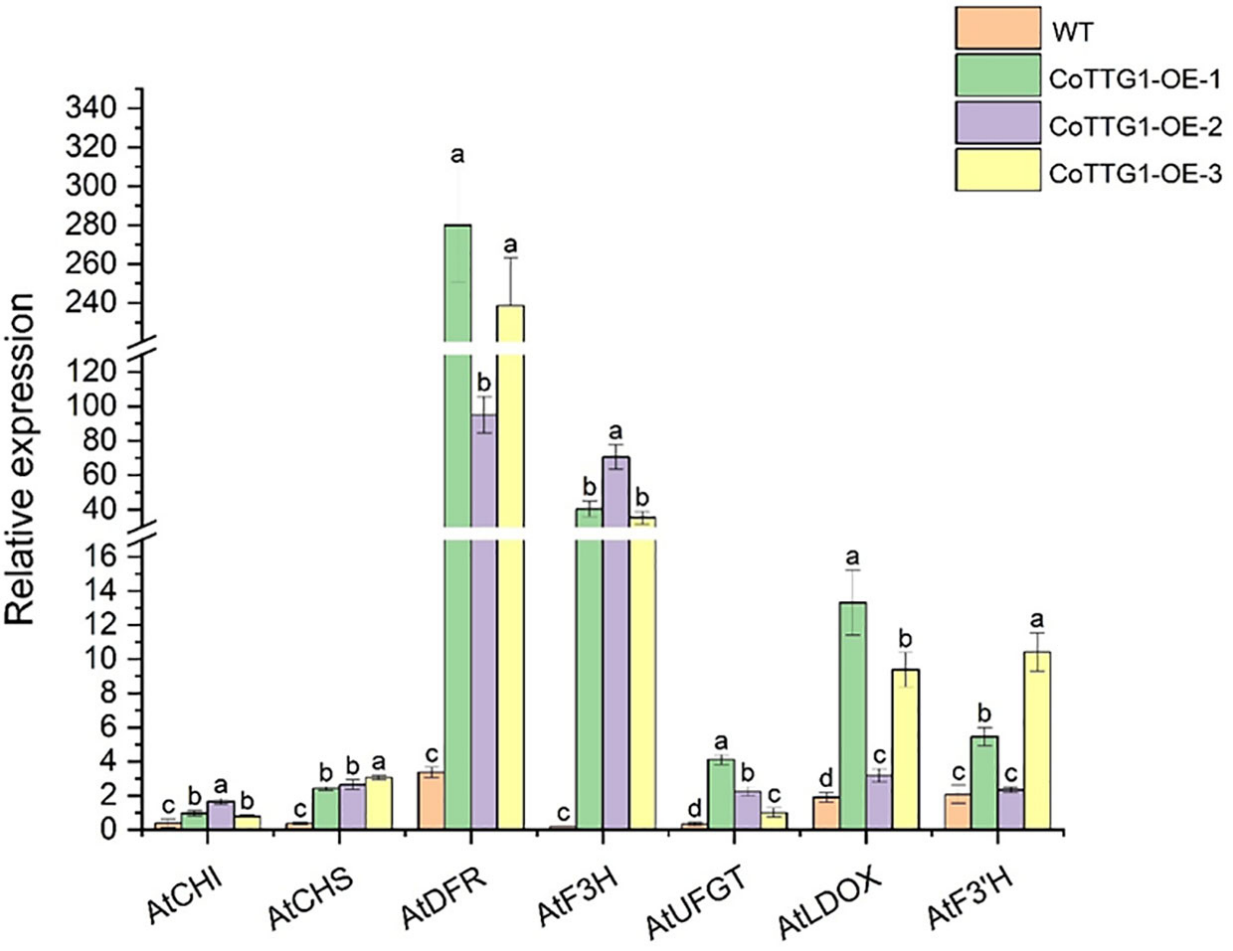
Quantitative analysis of the expression levels of genes related to seed and anthocyanin synthesis in transgenic plants. Letters indicate a significant difference at the p<0.05 level.

**Figure 12:**
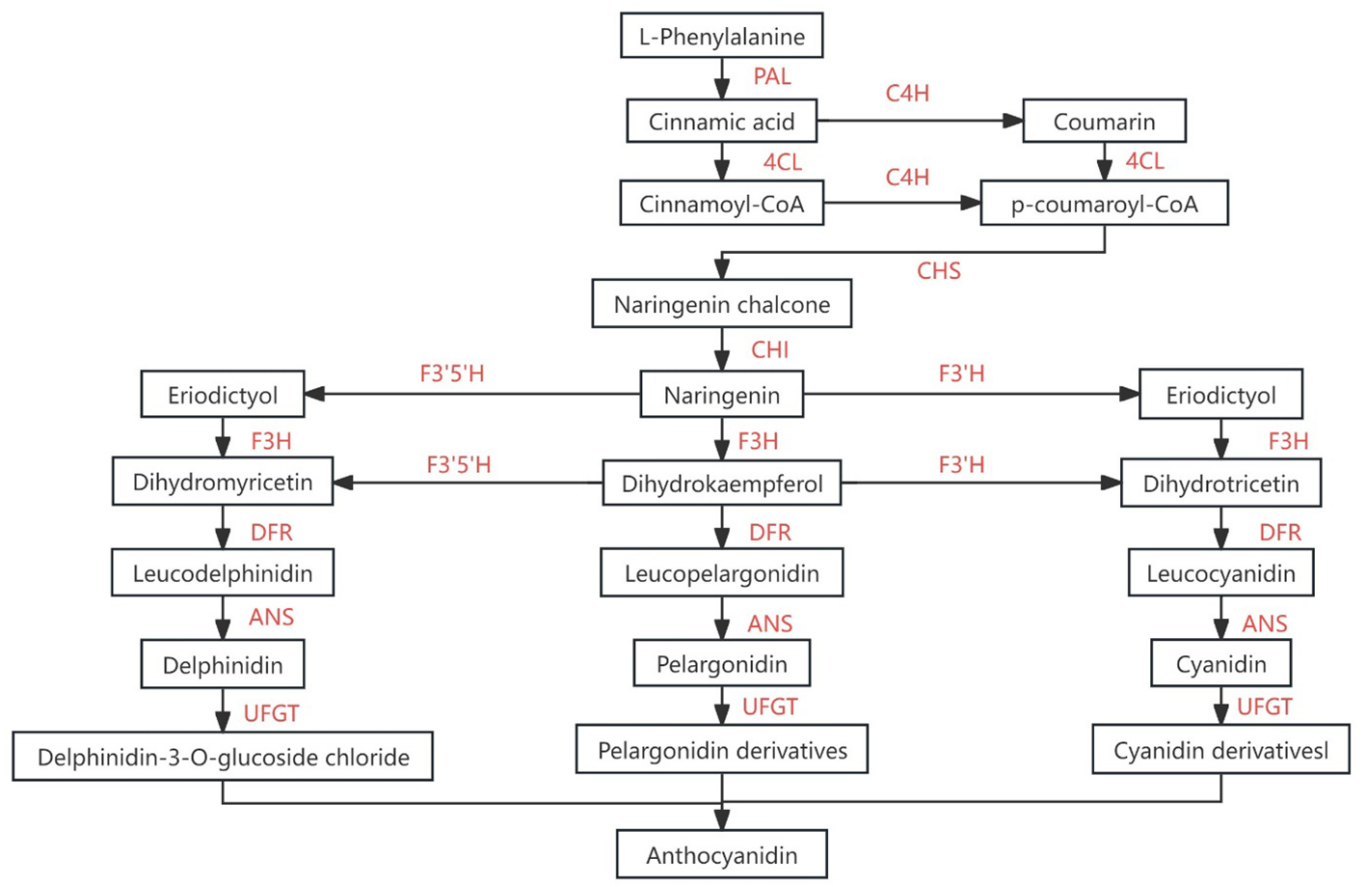
Flavonoids synthesis pathway [Adapted from Xu et al (Xu et al., 2015)]. *PAL*, *L-phenylalanin ammo-nialyase*、*C4H*, *cinnamicacid-4-hydroxylase*、*4CL*, *4-coumarate-Coligase*、*CHS*, *chalcone synthase*、 *F3H*, *favonoid-3-hydroxylase*、 *F’3H*, *flavonoid-3’-hydroxylase*、*LDOX (ANS)*, *anthocyanidinsynthase*、*UFGT*, *UDP- glucosyltransferase*.

The expression levels of genes related to anthocyanin synthesis were detected in leaves, flowers and seeds of transgenic plants. The results of quantitative PCR (qPCR) (Livak and Schmittgen, 2001) showed that the expression levels of *AtF3’H*, *AtUFGT* and *AtLDOX*, the key genes downstream of anthocyanin synthesis pathway, were significantly up-regulated in the leaves of transgenic plants compared with wild type (**Figure 9**). The expression of related genes upstream of the anthocyanin synthesis pathway, such as *AtCHS*, *AtCHI*, *AtF3H* and *AtDFR*, did not change significantly (**Figure 9**). In flowers, the expression of *AtCHS*, *AtCHI*, *AtLDOX*, *AtF3H*, and *AtUFGT* was significantly down-regulated (**Figure 10**); however, in seeds, their expression was markedly up-regulated (**Figure 11**). The expression of *AtCHI*, *AtCHS*, *AtDFR*, *AtF3H*, *AtUFGT*, *AtLDOX* and *AtF3’H* increased significantly in fruit (**Figure 12**). This 'seed-leaf-flower' differential regulation pattern (**Figure 12**) suggest that *CoTTG1* gene may effectively promote the accumulation of anthocyanins in *Arabidopsis* plants by up-regulating the expression levels of downstream key enzyme genes (such as *AtF3’H*, *AtLDOX* and *AtUFGT*) in the anthocyanin synthesis pathway, and play a regulatory role in the late stage of anthocyanin synthesis.

### 3.9 Analysis of total fatty acid content and components

**Figure 13:**
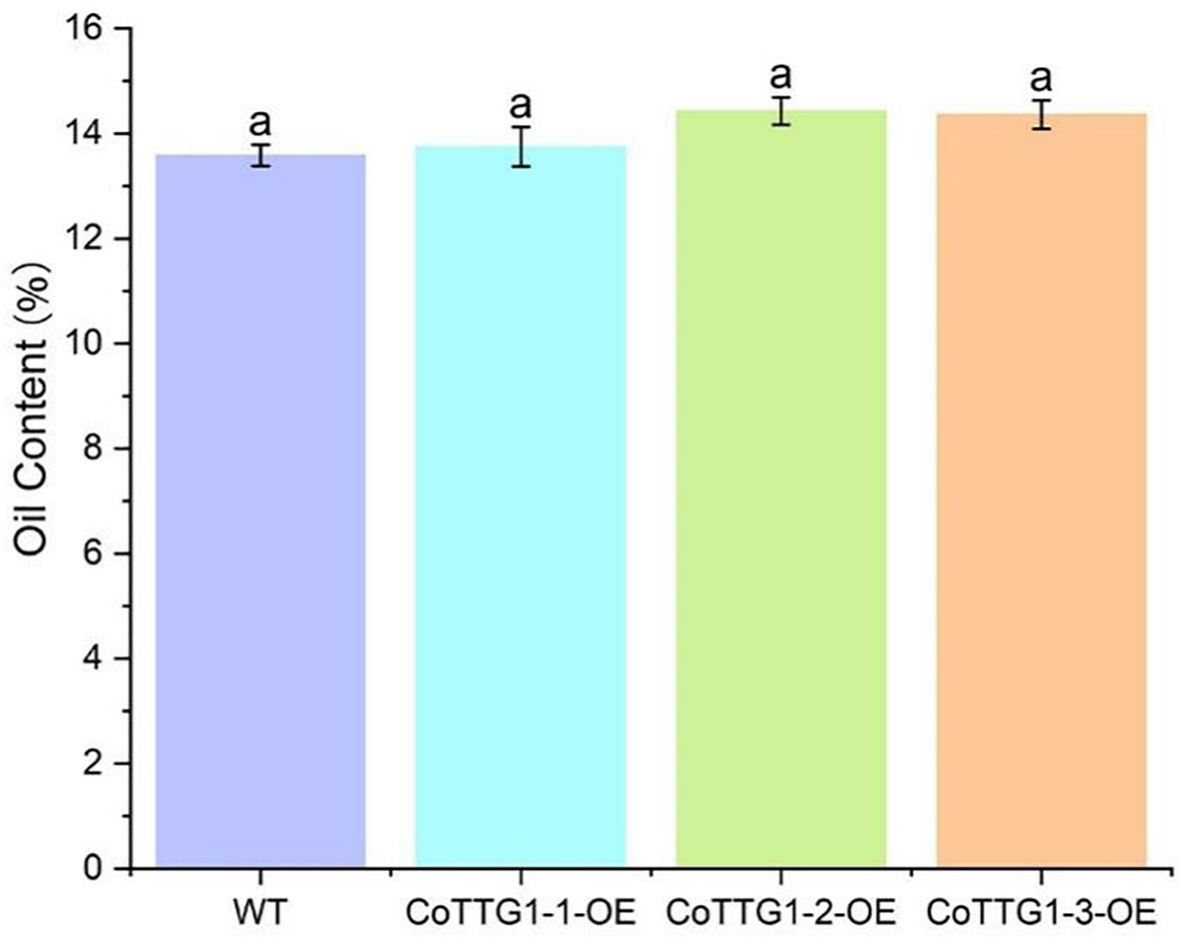
Oil content of mature *CoTTG1* transgenic *Arabidopsis* seeds. Letters indicate a significant difference at the p<0.05 level.

**Figure 14:**
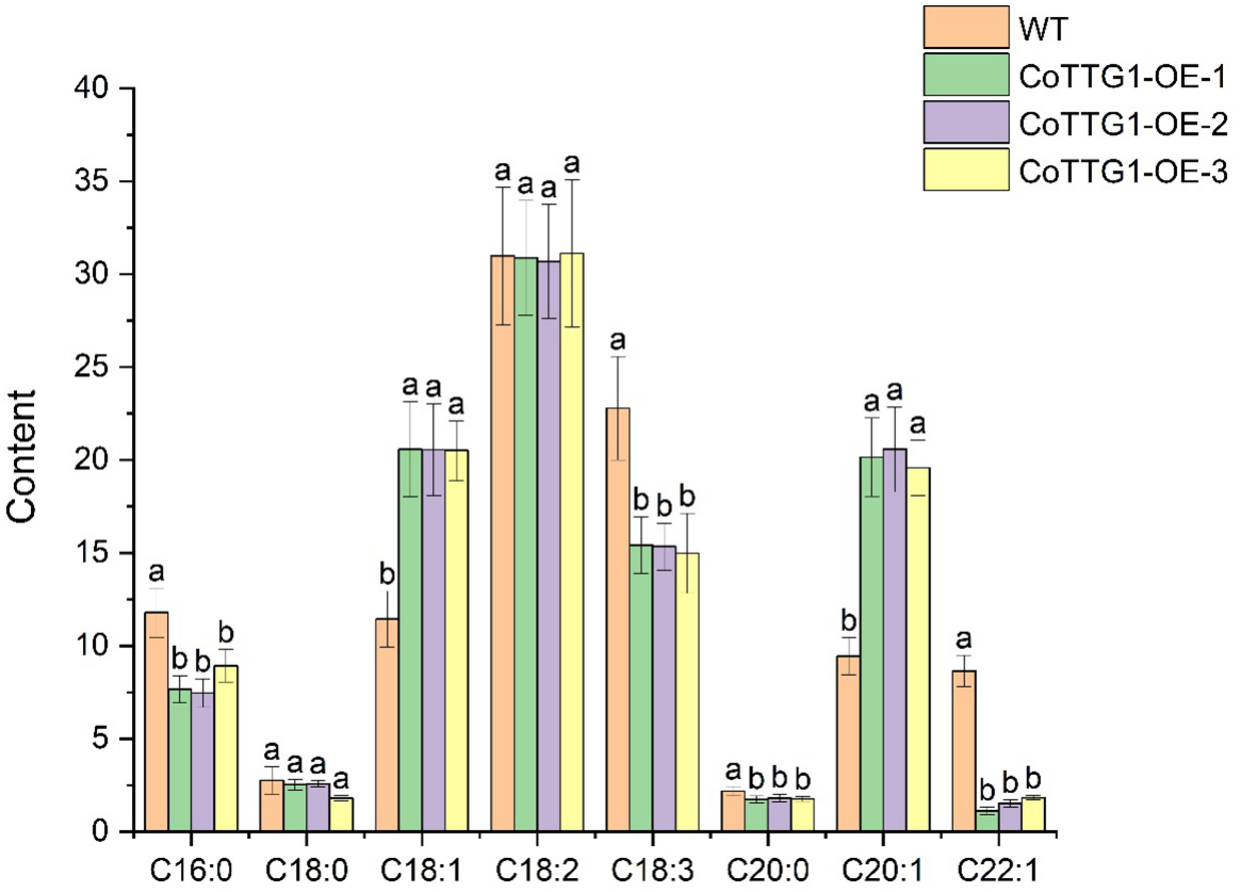
Analysis of oil component content in seeds of overexpression lines. C16:0 is palmitic acid, C18:0 is stearic acid, C18:1 is oleic acid, C18:2 is linoleic acid, C18:3 is linolenic acid, C20:0 is arachidic acid, C20:1 is Gong duo acid, C22:1 is erucic acid. Letters indicate a significant difference at the p<0.05 level.

The total fatty acid content in the seeds of *CoTTG1* overexpression transgenic *Arabidopsis thaliana* and wild-type *Arabidopsis thaliana* was shown in **Figure 10**. The seed oil content of *CoTTG1* overexpression lines showed no significant difference from the wild type, remaining at approximately 14% (**Figure 13**). Oleic acid (C18:1) and Gondoic acid were significantly increased by 79% and 113% in the seeds of *CoTTG1* overexpression lines (**Figure 14**). On the contrary, the contents of palmitic acid (C16:0), linolenic acid (C18:3) and erucic acid (C22:1) were significantly reduced by 35%, 35% and 87% in the seeds of *CoTTG1* overexpression lines (**Figure 14**). These results indicate that the overexpression of *CoTTG1* leads to a significant change in the content of fatty acid components, and has different effects on different fatty acid components.

### 3.10 Expression analysis of genes related to fatty acid composition

**Figure 15:**
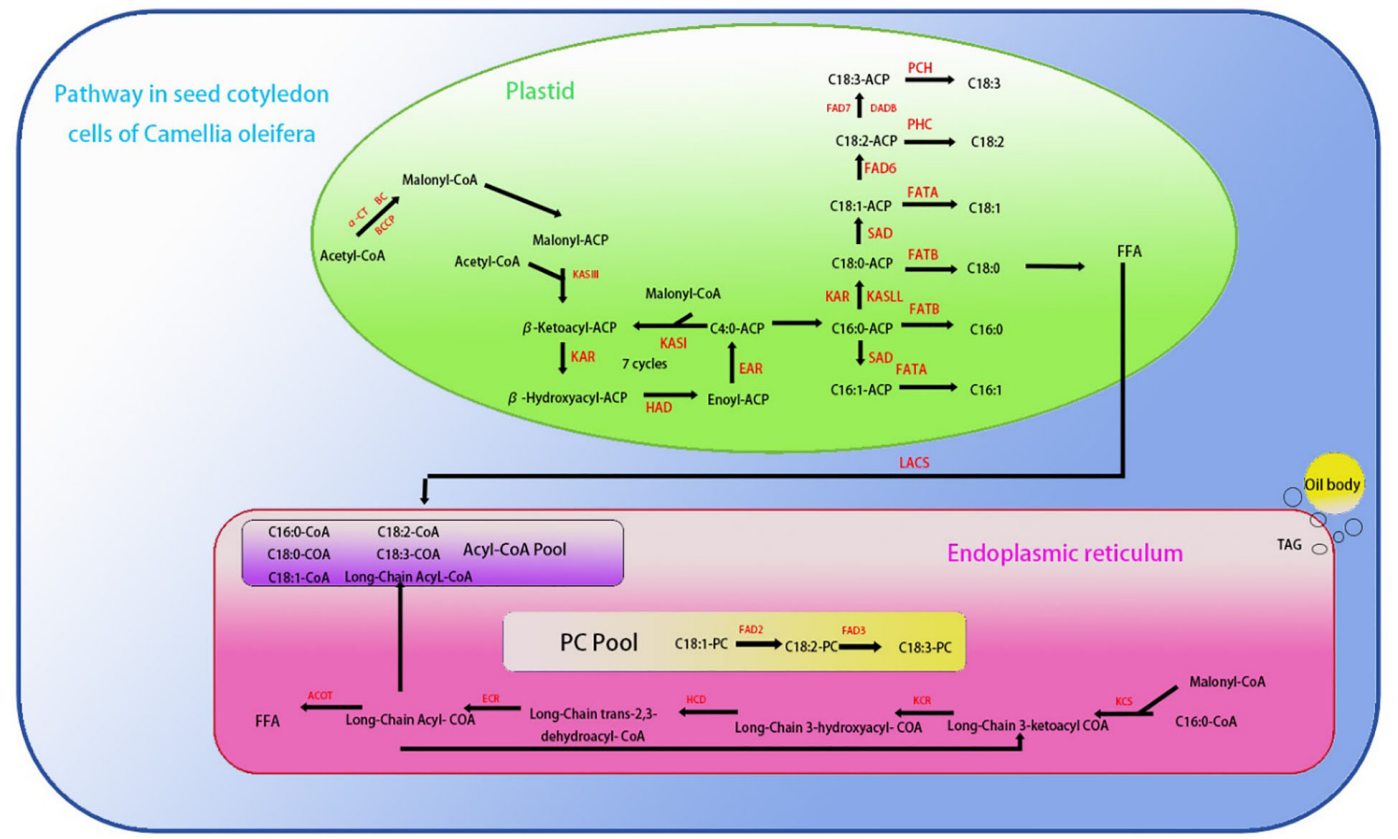
Saturated fatty acid and unsaturated fatty acid biosynthetic pathways in *Camellia oleifera* [Adapted from Xiaofeng et al (Xiaofeng, 2023)]: *KCS1, 3-Ketoacyl-CoA synthase 1、ACOT, Acyl-CoA thioesterase、KCR:3-Ketoacyl-CoA reductase、HCD, Hydroxy-CoA dehydratase、ECR, Enoyl-CoA reductase、FAD2:Fatty acid desaturase 2、FAD3:Fatty acid desaturase 3、FAD8:Fatty acid desaturase 8、FAD7:Fatty acid desaturase 7、FAD6:Fatty acid desaturase 6、PCH, Palmitoyl-CoA hydrolase、FATA, Fatty Acid TrANSporter A*.

**Figure 16:**
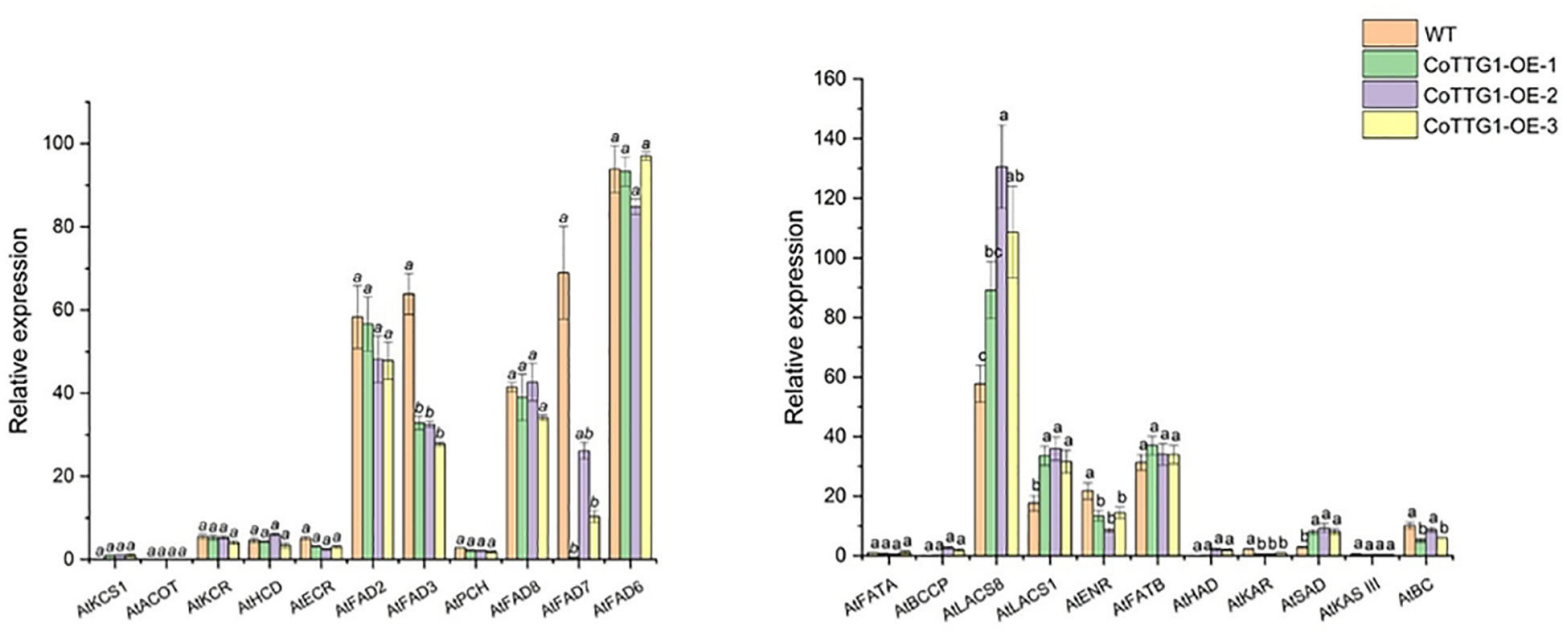
Expression analysis of genes related to fatty acid composition formation in seeds of overexpressing plants. Letters indicate a significant difference at the p<0.05 level.

Compared with wild-type plants, the expression levels of *AtLACS8*, *AtLACS1* and *AtSAD*, which are closely related to fatty acid composition, showed a significant upward trend in overexpression transgenic plants (**Figure 15**). These three genes are mainly involved in catalyzing the conversion of long-chain fatty acids into their coenzyme A derivatives (Acyl-CoA) and catalyzing the conversion of stearic acid (C18:0) into oleic acid (C18:1) components (Baud et al., 2002). Among them, the expression level of *LACS8* gene increased the most, reaching 89% (**Figure 15**). *AtENR*, *AtBC* and *AtFAD3* showed a significant downward trend, mainly involved in the reduction of olefin structure after dehydrogenation, acyl-ACP and acetyl-CoA (Acetyl-CoA) to form new long-chain fatty acids and linoleic acid (C18:2). Further conversion to linolenic acid (C18:3) components, *AtFAD3* gene decreased the most significantly, decreasing by about 51% (**Figure 15**). The increased expression of these genes was consistent with the change trend of corresponding fatty acid components in seeds. The above results indicate that the *CoTTG1* gene in *C. oleifera* is likely to effectively change the formation of fatty acid components in seeds by enhancing the gene expression levels of *AtLACS8*, *AtLACS1* and *AtSAD* (**Figures 16**). Gene expression analysis confirmed CoTTG1's impact on fatty acid desaturases (FAD3) and other key lipid metabolism genes.

## 4 Discussion

### 4.1 *CoTTG1* is an evolutionarily conserved WD40 protein

WD40 protein is a large family in eukaryotes and plays a key role in a variety of biological processes, such as signal transduction, transcriptional regulation, cell cycle regulation and apoptosis.

The *CoTTG1* gene sequence was successfully cloned and obtained by screening the whole genome of *C. oleifera* combined with transcriptome data. The protein structure analysis showed that the amino acid sequence of *CoTTG1* was highly similar to *Camellia* and *Theaceae*, reaching 99.71% and 99.51%, respectively. The similarity with other species is more than 80%, which indicates that *TTG1* is highly conserved in protein sequence structure. Through the comprehensive analysis of the phylogenetic relationship of *CoTTG1* gene in the WD40 gene family and its phylogenetic relationship among *TTG1* genes of different species, it is concluded that the *TTG1* gene in *C. oleifera* is not a product of accidental mutation, but gradually developed in strict accordance with the phylogenetic evolution process among species. *TTG1* gene has maintained a high degree of structural similarity and functional conservation during evolution, which further highlights its important position and role in plant growth and development. Despite high conservation in protein sequence, *TTG1* achieves functional diversity through dynamic combinations of its WD40 domain with tissue-specific MYB and bHLH transcription factors. In *Arabidopsis*, *TTG1* regulates distinct physiological processes by forming organ-specific MBW (MYB-bHLH-WD40) complexes: activating proanthocyanidins biosynthesis via TT2-TT8 in seed coats, promoting anthocyanin accumulation through PAP1-TT8 in leaves, and controlling trichome development by interacting with GL1-GL3 in epidermal cells (Walker et al., 1999; Baudry et al., 2004; Gonzalez et al., 2008) This pleiotropic nature suggests that *TTG1*’s functional conservation relies not only on sequence preservation but more crucially on its combinatorial partnerships within specific tissue contexts. Our findings that *CoTTG1* shows peak expression during early seed development and alters fatty acid profiles in transgenic *Arabidopsis* imply its potential involvement in *C. oleifera* quality traits through analogous mechanisms, though the precise interaction networks warrant further investigation.

### 4.2 Expression pattern of *CoTTG1* gene

The expression level of *TTG1* gene in *C. oleifera* seeds showed significant dynamic changes during development. From July, its expression level gradually increased, and continued to decline after reaching its peak in September. At the same time, the oil content of seeds was close to zero in mid-July. With the development of seeds, the oil content increased significantly from mid-September to early October, and entered the platform period after October. Correlation analysis showed that the expression peak of *TTG1* gene (September) was earlier than the rapid accumulation period of oil (late September to early October), suggesting that the gene may play a regulatory role in the upstream of the oil synthesis pathway. However, although the gene expression level was positively correlated with the oil content, no significant change in the oil content was observed in the *TTG1* overexpression plants. This contradiction may be due to the following mechanism: the regulation of *TTG1* may be limited by the endogenous expression threshold, the complexity of the multi-gene collaborative regulatory network, or its function depends on the post-translational modification and protein interaction environment at a specific developmental stage.

This study found that the *CoTTG1* gene showed a developmental stage-specific expression pattern in transgenic *Arabidopsis*, especially in the early stage of seed development (pod stage 4). Subcellular localization results showed that *CoTTG1* protein was localized in the nucleus, suggesting that it may play a role as a core component of the transcriptional regulatory complex. Similar to *Arabidopsis AtTTG1* (Walker et al., 1999), it is speculated that *CoTTG1* may form different protein complexes by recruiting different bHLH and MYB transcription factors to regulate biological processes such as trichome development, anthocyanin synthesis and fatty acid metabolism (Zhao et al., 2008). These findings not only enrich the understanding of the functional diversity of *TTG1*-like genes, but also provide a new theoretical basis for the quality improvement of economic crops. While our bulk tissue analyses revealed *CoTTG1*’s regulatory roles, it should be noted that trichomes constitute less than 2% of leaf cells (Shulse et al., 2019), and anthocyanin/fatty acid metabolism may occur in specialized cell types. The mixed-tissue approach could mask cell-specific expression patterns. Future single-cell RNA-seq or laser microdissection studies would help resolve these spatial dynamics.

### 4.3 Functional verification of *CoTTG1* gene

#### 4.3.1 *CoTTG1* gene regulates trichome formation

R2R3 MYB transcription factors *GL1*, *MYB23*, *MYB82*, bHLH transcription factor *GL3*, enhancer *EGL3* and WD40 repeat protein *TTG1* constitute the core regulatory network of trichome formation (Liu et al., 2021). Studies have shown that *TTG1* is actively involved in the formation and development of trichomes by interacting with *GL1* and *GL3*/*EGL3*. The GL1-GL3/EGL3-*TTG1* complex can also further affect the formation of trichomes by inducing the expression of *GL2* and some single-repetitive R3 MYB transcription factors (Walker et al., 1999). This regulatory process covers both positive and negative transcription factors, forming a complex and closely linked regulatory network, which plays a decisive role in the development of plant trichomes. In the overexpression lines of *CoTTG1* gene, the expression of *AtGL1* gene was significantly up-regulated, which promoted the development of *Arabidopsis* trichome. In addition, it was also found that the expression levels of *AtETC1* and *AtCPC*, inhibitors of trichome formation, were also increased. It is speculated that the surge in *GL1* expression may break the balance of the original trichome regulatory network. The increased expression of inhibitory genes such as *AtETC1* and *AtCPC* did not counteract the positive regulation of *AtGL1*, suggesting the existence of unknown feedback mechanisms in the trichome regulatory network that maintain developmental balance.

Notably, trichomes constitute <2% of leaf cells (Shulse et al., 2019; Zhang et al., 2019), suggesting *CoTTG1*’s dramatic effects may involve both direct gene activation (e.g., *GL1*) and systemic hormone regulation.

#### 4.3.2 *CoTTG1* gene regulates anthocyanin synthesis

The study found that the peak expression of *LhTTG1* gene in the flowering process of *Lilium* spp. *occurs* at the coloring stage, which is highly consistent with the critical period of anthocyanin accumulation (Zhao, 2007). The expression pattern of *IuTTG1* gene showed a significant flower color correlation in different flower color varieties of Impatiens *uliginosa*. The expression level of *IuTTG1* gene in deep red varieties was significantly higher than that in white varieties (Tan et al., 2024). In this study, heterologous overexpression of the *CoTTG1* gene resulted in a deeper reddish-brown seed coat and a significant increase in anthocyanin content in seeds, indicating that *CoTTG1* may be a structural enzyme or regulator involved in the proanthocyanidins synthesis pathway. *TTG1* in other species also has the ability to regulate anthocyanin and proanthocyanidins biosynthesis.

The biosynthesis of flavonoids has always been a research hotspot. The structural genes and enzyme genes involved in the process have been widely identified and studied (Carey et al., 2004). The accumulation of anthocyanin in plants is mainly controlled by the changes of key genes at the transcriptional level (Ban et al., 2007). In a large number of studies on anthocyanin synthesis, two key transcription factor families, bHLH and R2R3 MYB proteins (Mehrtens et al., 2005), have been found. The MBW (MYB-bHLH-WD40) complex composed of them and WD40 transcription factors plays a crucial regulatory role in flavonoid accumulation (Tanaka and Ohmiya, 2008). As an important member of the WD40 family, *TTG1* plays a key activation role in this process. At present, it is not clear which type of transcription factors interact with *TTG1* in *C. oleifera* to form a complex, which needs further study. In this study, the expression of key genes in the anthocyanin synthesis pathway in *CoTTG1* overexpression lines was detected. Overexpression of *CoTTG1* significantly increased the expression levels of *CHI, CHS, DFR, F3H, F’3H, LDOX* and *UFGT* in seeds, among which *DFR* increased the most, by nearly 60%. In leaves, overexpression of *CoTTG1* mainly promoted the expression of *F’3H* and downstream genes *LDOX* and *UFGT*. The expression of *F3H* and *UFGT* in flowers was also inhibited. This indicates that there are some differences in anthocyanin synthesis between flowers and leaves and seeds. In seeds, the expression level of *DFR* increased most significantly (60%). DFR catalyzes the conversion of dihydroflavonol to colorless anthocyanins, and its enhanced expression may directly lead to the accumulation of anthocyanin precursors in seeds (Li et al., 2019). At the same time, the expression of *CHS* (*chalcone synthase)* as a key enzyme for the initiation of the synthesis pathway was up-regulated (Li et al., 2019), suggesting that *CoTTG1* may simultaneously enhance precursor supply and branch conversion efficiency through a ‘ two-pronged ‘ strategy. However, in floral organs, the expression of *F3H* and *UFGT* is inhibited, which may be a negative feedback regulation mechanism specific to flower tissue. When the anthocyanin accumulation reaches the threshold, the homeostasis is maintained by inhibiting the expression of synthase. *LDOX* (*leucine aminopeptidase*) is specifically activated in leaves, which catalyzes the conversion of colorless anthocyanins to colored anthocyanins. Its tissue-specific expression pattern may explain the changes in specific anthocyanin components observed in leaves. The expression changes of these genes showed a ‘seed-leaf-flower’ triple differential regulation pattern, suggesting that *CoTTG1* may form a tissue-preferred regulatory complex by combining with different tissue-specific MYB/bHLH factors (such as TT2-TT8 in seeds, PAP1-GL3 in leaves, etc.).

#### 4.3.3 *CoTTG1* regulates oil synthesis and fatty acid composition

Plant oil content is a quantitative trait regulated by multiple genes, and the expression of genes determines the rate of oil accumulation in plant seeds. At present, more than 600 genes related to *Arabidopsis* oil synthesis have been identified in the *Arabidopsis* genome, including 19 genes related to seed oil storage (Liu et al., 2022). In this study, there was no significant change in the oil content of seeds in transgenic *Arabidopsis* overexpressing *TTG1*, and there was no significant change in the expression of *AtKCS1, AtKCR, AtHCD, AtECR, AtACOT* genes related to lipid synthesis. The effect of *CoTTG1* on lipid synthesis is not obvious, and its effect on lipid may be reflected in other aspects.

In addition, the fatty acid composition of the seeds of *CoTTG1* transgenic lines was analyzed. The results showed that the fatty acid composition changed significantly compared with wild-type *Arabidopsis thaliana*. The content of oleic acid (C18:1) and Gongduo acid (C20:1) increased significantly, while the content of palmitic acid (C16:0), linolenic acid (C18:3) and erucic acid (C22:1) decreased significantly. These changes suggest that *CoTTG1* transgenic may affect the composition of fatty acids in seeds by regulating key genes in the fatty acid synthesis pathway.

Further analysis of the expression of genes related to fatty acid synthesis showed that the expression levels of *AtEAR* and *AtFAD3* genes decreased, while the expression levels of *AtLACS8*, *AtLACS1* and *AtSAD* genes increased. These changes may be the key factors leading to the transformation of fatty acid composition. In particular, the decreased expression of *AtFAD3* gene may be the reason for the decrease of linolenic acid (C18:3) content, while the up-regulation of *AtLACS8*, *AtLACS1* and *AtSAD* genes may promote the accumulation of oleic acid (C18:1) and tributyrate (C20:1).At the same time, the up-regulation of *SAD* gene may play an important role in the conversion of stearic acid (C18:0) to oleic acid (C18:1). For example, in the dynamic analysis of fatty acid accumulation and related gene expression in *C. vietnamensis*, it was found that the enhanced expression of *SAD* gene significantly promoted the conversion of stearic acid to oleic acid (Guo et al., 2020). In addition, the study of sesame stearoyl-ACP desaturase gene *SiSAD* also showed that *SiSAD* positively regulated the increase of oleic acid content in the process of oleic acid synthesis and metabolism (Zhou et al., 2019). Therefore, the up-regulation of *SAD* gene may be an important factor leading to the increase of oleic acid content in *CoTTG1* transgenic lines. *ECR* plays an important role in the synthesis of palmitic acid (C18:0). It helps to synthesize saturated fatty acids from unsaturated fatty acids such as linoleic acid (C18:2) or oleic acid (C18:1) by reducing olefin chains, including palmitic acid (Haslam and Kunst, 2013). The up-regulation of *ECR* activity will increase the synthesis of palmitic acid, and if the expression of *ECR* is inhibited, the accumulation of palmitic acid will be reduced. *FAD3* gene is a key gene for seed linolenic acid synthesis, and its expression is regulated by multiple transcription factors. Transcription factors regulate the expression of *FAD3* gene by interaction and determine the content of α-linolenic acid in seeds (Yang et al., 2023). When the expression of *FAD3* decreased, oleic acid (C18:1) could not be effectively converted into linolenic acid, resulting in a decrease in linolenic acid content. It is worth noting that although the expression of some genes related to fatty acid synthesis, such as *AtKCS1, AtKCR, AtHCD, AtECR* and *AtACOT*, did not change significantly, indicating that there may be potential gene regulatory pathways that have not been fully revealed. These pathways may play an important role in the changes of fatty acid composition in *CoTTG1* transgenic lines.

## 5 Conclusions

In this study, the *CoTTG1* gene of *C. oleifera* was successfully identified, which encodes a WD40 repeat protein located in the nucleus and plays a central role in regulating a variety of important agronomic traits. The study found that overexpression of *CoTTG1* in *Arabidopsis* significantly increased trichome density, indicating that the gene has a conserved regulatory function in trichome development. *CoTTG1* promotes anthocyanin accumulation in seed coat, revealing its key role in the regulation network of secondary metabolism. This gene also affects the composition of fatty acid components, confirming the pleiotropic effect of *TTG1* family genes in the synthesis of storage substances. These findings not only elucidate the multiple regulatory mechanisms of *CoTTG1* in trichome formation, anthocyanin synthesis and fatty acid metabolism, while future cell-type-specific studies could further resolve its spatial regulation mechanisms, but also provide important target genes and theoretical basis for molecular breeding to improve appearance quality (trichome) and nutritional value (fatty acid composition) in economic crops such as *C. oleifera*.

## Data Availability

The datasets presented in this study can be found in online repositories. The names of the repository/repositories and accession number(s) can be found in the article/**Supplementary Material**.
